# Comparison of Endodontic Treatment Results Yielded from Using Normal Saline with IKI Final Rinse or NaOCl Irrigation: A 30-Month Follow-up Study

**Published:** 2013-10-07

**Authors:** Abbas Abbaszadegan, Mohammadreza Nabavizadeh, Ameneh Hoseini Yekani, Akbar Khayat

**Affiliations:** a Department of Endodontics, Dental School, Shiraz University of Medical Sciences, Shiraz, IR Iran; b Department of Dental Public Health, Shiraz University of Medical Sciences, Shiraz, IR Iran; c Division of Endodontics, Department of Oral Biological and Medical Sciences, Faculty of Dentistry, University of British Columbia, Canada

**Keywords:** Apical Periodontitis, Healing, Iodine Potassium Iodide, Sodium Hypochlorite

## Abstract

**Introduction:**

The aim of this clinical trial was to evaluate and compare the endodontic treatment results of teeth with apical periodontitis after thirty-month recall with two different irrigation regimen: normal saline followed by Iodine Potassium Iodide (IKI) or sodium hypochlorite (NaOCl) irrigation alone.

**Materials and Methods:**

Twenty seven patients (30 teeth) who had been included in the first part of our antimicrobial survey were recalled. In previous stage, root canal treatments were performed using either normal saline with IKI final rinse (n = 15) or NaOCl (n = 15) as irrigating solutions. Bacterial samples were taken before and after instrumentation. In this stage, three patients (6 teeth) were excluded from the follow-up schedule since they did not respond to the recall requests. The remaining 24 subjects (12 teeth in each NaOCl and IKI group) were examined clinically and radiologically. Post-operative and follow-up images were coded, blindly evaluated and given a periapical score according to PAI scoring system. The outcome was assessed in two ways; first, the changes in PAI score from base line to the follow-up evaluation in each group were assessed by wilcoxon signed rank test. In addition, Mann-Whitney U test was used to compare the differences between the post-operative and follow-up images of treatment groups. Second, the dichotomous variables as “healed” or “not healed” were compared.

**Results:**

The Median (Min, Max) PAI scores for NaOCl group and IKI group were both 5 (3, 5) for immediate post-operative radiographs and declined to 1 (1, 2) and 2 (1, 2), respectively. A statistically significant decrease in PAI score from the base line to the follow-up evaluation was seen in both groups (P = 0.002). The decrease in NaOCl group was higher significantly in comparison to IKI group (P = 0.036). One hundred percent of the teeth were healed in both groups (PAI ≤ 2) and no teeth showed any abnormal clinical findings.

**Conclusion:**

Root canal irrigation with NaOCl resulted in a significant higher decrease in PAI scores in comparison to irrigation with normal saline followed by IKI final rinse. Although, according to results of 30-months recall, complete bone formation was observed in all samples in both groups and no teeth showed any abnormal clinical findings. These findings depict the weight of all important clinical and biological factors which together impact the results of a successful endodontic treatment.

## 1. Introduction

Microorganisms and their metabolites are found to be the main aetiology for the pulp and periapical diseases (1). Hence, the endodontic treatment success critically depends on the degree of microbial control and the quality of treatment (2). Various intracanal irrigants can be used to minimize the bacterial load in the infected teeth. Two of these antimicrobial solutions are sodium hypochlorite (NaOCl) and iodine potassium iodide (IKI). Each of these irrigants has its own particular properties. NaOCl has the wide range antimicrobial activity but it has known to depict some clinical disadvantages as well. The major concern is about its cytotoxicity (3, 4). It may also alter

**Table 1.** PAI scoring system (adapted From Orstavik et al., 1986)

PAI Score	Radiographic findings
**1**	Normal periapical structures
**2**	Small changes in bone structure
**3**	Changes in bone structure with some mineral loss
**4**	Periodontitis with well-defined radiolucent area
**5**	Severe periodontitis with exacerbating features

the mechanical properties of both dentin and nickel titanium instruments (5-7).On the other side, IKI is a strong antimicrobial agent with acceptable and less undesirable properties in comparison with NaOCl (3, 4) but the evaluations regarding its antimicrobial efficacy reveal conflicting results. Some previous in vitro (8) and in vivo (9, 10) studies have reported the effectiveness of IKI as final rinse but some other clinical studies failed to report the benefits of its antimicrobial effect (11, 12). Moreover, there are no sufficient data on healing outcome after its use.

Apart from the effectiveness of any antimicrobial protocol, it’s apparent that short-term microbial investigations may not imply the long term outcome of endodontic treatments (13, 14). Many authors suggested one year follow-up to be an adequate period, (13-16) while the follow-up of 4 years may be required to display the constant healing status (14, 17, 18). Surveys evaluating the outcome should be based on both clinical and radiological appraisal (19, 20). Periapical index (PAI) is commonly used to avoid the inconsistency of radiologic assessment and also to standardize the evaluation processes. In this index, lower scores (scores 1 and 2) are considered as “healthy” and the higher scores (scores 3 to 5) are labeled as “unhealthy” regarding the status of periapical region (21). Bias in interpretation of radiographs will be minimized if blinded examiners provide the evaluations (21, 22).

The ratio of complete healing after initial treatment of the teeth with chronic apical periodontitis, ranges from 73% - 90% and this proportion can increase over the time (17, 23). In the first part of this in vivo study performed in infected teeth with apical periodontitis, we evaluated the antimicrobial efficacy of IKI final rinse following normal saline irrigation and compared it with NaOCl as an irrigant (12). The results revealed that irrigation with NaOCl was significantly more efficient in bacterial load reduction. Since the following up of treated patients is crucial for accurate evaluation of the efficiency of any employed antimicrobial irrigants; the second stage of the study was designed to compare and evaluate the healing of treated teeth of those patients after a period of thirty months.

## 2. Material and Methods

### 2.1. Follow-up examination

This study was approved by the Ethics Committee of Shiraz University of Medical Sciences # 98-5109 and was registered in the Iranian Registry of Clinical Trials website (www.irct.ir) with registration ID # IRCT138903264196N1. Study subjects had

**Table 2.** The baseline and the follow-up PAI scores of all cases in each group

	NaOCl		IKI	
Case no.	Baseline PAI	Follow-up PAI	Baseline PAI	Follow-up PAI
**1**	4	1	5	2
**2**	5	1	4	2
**3**	5	1	5	2
**4**	4	1	5	1
**5**	5	1	4	2
**6**	5	2	3	1
**7**	5	1	5	1
**8**	3	1	4	2
**9**	5	1	5	2
**10**	4	1	5	2
**11**	5	1	5	2
**12**	4	1	4	2

been selected from the patients presented to the endodontic clinic at Shiraz School of Dentistry. In previous stage, thirty single-rooted teeth with necrotic pulps in 27 patients were selected according to specific inclusion and exclusion criteria and divided into two random groups. In group I, canals were irrigated with 2.5% NaOCl during instrumentation and in group II, canals were initially irrigated with sterile saline during biomechanical preparation and then exposed to a 5-minute final irrigation with 2% IKI (iodine, 2 %; potassium iodide, 4%; and distilled water, 94%). Bacterial samples were taken before treatment and at the end of treatment. Then the teeth were obturated in the same session. The obturation was performed using gutta-percha (Dentsply Maillefer, Tulsa, OK) and Tubli-Seal sealer (ZOE-based; SybronEndo, Orange, CA, USA) by lateral condensation technique.

In this stage, all 27 patients (30 teeth) who had been participated in previous study were recalled by phone in 30 months after treatment. We encouraged the participants to attend a follow-up examination by offering discount for their future dental treatments. The primary outcome measure for this study was any increase or decrease in the apical radiolucency. The secondary outcome measures were the presence or absence of any sign and symptoms and the proportion of healed teeth in each group.

Patients were examined clinically and radiographically. Presence of sinus tract, swelling, tenderness to percussion or palpation and probing depth greater than the baseline measures were recorded by an investigator who was unaware of the purpose of the study. Teeth with extreme caries, root fractures and with leakage in restorations were excluded. The follow-up radiographs were made with same measures and exposure settings as with immediate postoperative images using the parallel technique and a FOCUS X-ray intraoral unit (Focus, Instrumentarium Dental, Tuusula, Finland) with a XCP holder (Rinn Corp, Elgin, IL, USA).

**Table 3.** Median (Min, Max) PAI scores of each group at immediate postoperative and 30-month evaluation

Groups	Median (Min, Max)		P-value ^a^	Median of change	P-value ^b^
	Baseline PAI	Follow-up PAI			
**NaOCl group**	5 (3, 5)	1 (1, 2)	0.002	-3.5	0.036
**IKI group**	5 (3, 5)	2 (1, 2)	0.002	-3.0	

^a^ Wilcoxon signed rank test.

^b^ Mann-Whitney U test.

For the purpose of calibration, a series of radiographs (not related to the study samples) which were representing a varity of periapical bone densities were graded by three experienced endodontists before evaluation. These calibrated and blinded examiners were invited to analyze then scrutinize the radiographs of the study samples. The radiographic images were coded. Post-operative and follow-up images were evaluated randomly in a dark room with aid of a radiograph view box and magnifying glass and were given a periapical score according to PAI scoring system (Table 1). This procedure was repeated again after one month to check the intra-rater reliability. 

Disagreement between endodontists were resolved by several re-evaluations and reviewing the scores to reach the final consensus. Intra-rater and inter-rater reliability test (kappa coefficient) was performed to assess the interpreters’ agreement. The guidelines for assessment of the agreement proposed by Landis and Koch (24) were used as follow: 0.00-0.20, slight agreement; 0.21-0.40, fair agreement; 0.41-0.60, moderate agreement; 0.61-0.80, substantial agreement and 0.81-1.00 almost perfect agreements.

The outcome was assessed in two ways. First, Mann-Whitney U test was used to compare the differences between the post-operative and follow-up images of treatment groups. Wilcoxon signed rank test was conducted to examine the changes in PAI score from base line to the follow-up evaluation in each group. Second, dichotomous variables (PAI ≤ 2 assumed as “healed” and PAI > 3 as “not healed”) between NaOCl and IKI groups were compared.

### 2.2. Sample size and power determination

Since the study subjects had been previously recruited, it was not possible to determine the sample size for the current study. Instead, we calculated the post-power value based on the results with the method described by Walter (25) for non-parametric Mann-Whitney U test. All statistical analyses were performed using SPSS version 15.0 for Windows (SPSS Inc, Chicago, IL). P < 0.05 was considered as statistically significant.

## 3. Results

Three patients (6 teeth) were excluded from the follow-up since they did not respond to the recall requests. The remaining 24 teeth (12 teeth in each NaOCl and IKI group) were consisted of 8 upper central incisors, 5 upper lateral incisors, 4 upper second premolars, 6 lower premolars and 1 lower canine. The mean age of participants (7 man and 17 women) was 28 ± 9.7 and ranged from 18 to 42 years.

The recall rate was 80%. There was no statistically significant difference between groups, considering the age, sex and tooth location (P = 1). The level of agreement between endodontists was substantial (0.71 < k < 0.78). The Median (Min, Max) PAI scores for NaOCl group and IKI group were both (3, 5) for immediate post-operative radiographs and were declined to (1, 2) and (1, 2) respectively after the 30-month follow-up. A statistically significant decrease in PAI score was seen in both groups (P = 0.002), however, the decrease in NaOCl group was higher significantly (P = 0.036). The baseline and the follow-up PAI scores of all cases in each group were shown in Table 2. The statistical analyses of the results were summarized in Table 3. 

Regarding the secondary outcome measures, no teeth showed any abnormal clinical findings at 30-month recall evaluation. Besides, complete bone formation was observed in all samples in both groups and one hundred percent of the teeth were considered as healed (PAI ≤ 2).

## 4. Discussion

According to Strindberg criteria (26), the endodontic treatment outcome depends on the absence or the presence of apical periodontitis. However; delayed disappearance of apical radiolucency should not be considered as a “failure" whilst the involved teeth are in healing phase and are functional (14, 27). Although in non-surgical root canal treatment the complete healing may require several years to be taken place (26, 28), studies showed that shorter time would also be fair enough to observe meaningful changes (16, 29, 30).

This clinical trial study was designed to assess the periapical healing of the treated teeth in our previous study after a 30-month recall. In the first stage, 30 single-rooted teeth with apical periodontitis were irrigated either with NaOCl or normal saline followed by IKI as a final rinse. All treatment procedures were performed with the same method for both groups by one endodontist. The quality of the root canal treatments and coronal restorations were within the acceptable standards. The participants were 7 men and 20 women aged 20 to 64 years old (12). In the second stage, six patients were dropped out and the remaining 24 patients were presented for follow-up examinations. The dropout is a normal issue for follow-up evaluations especially where a treatment option is meant to be appraised, but fortunately it resulted in an equal amount of cases in both groups. Although, the limited sample size is similar to the other enrolled and published studies (19, 31, 32), the recall rate of 80 % seems to be rational to attain a sufficient level of evidence (33).

The primary and secondary outcome assessment strategies for this study were the radiological evaluation accompanying by clinical appraisal. To minimize the possible bias and inconsistency, three experienced endodontists were involved to interpret the coded radiographs blindly by PAI scoring system at two separate times (34). The kappa statistic was calculated and ranged from 0.71 to 0.78, indicating substantial agreement. Clinical examinations were performed by an independent examiner who was not involved in the treatment processes.

Since we had to measure the dependent variable of this study (healing) in an ordinal scale (score:1-5) and the assumption of normality was not reasonable, we used the nonparametric Mann-Whitney U test to compare the medians of differences between the groups. However, some previous studies considered the PAI scores as a set of data with normal distribution and employed parametric methods for their statistical analyses (34, 35).

As the sample size of this study was predetermined by the previous phase, we calculated the post-power value, considering the current sample size, based on the yielded results. In this respect, an 80.8% post-power value was obtained at α = 0.05 level of significance which implies that the number of cases was sufficient to obtain significant results.

The results showed no link between the treatments outcome of 2 study groups with age and gender of the participants. Besides, as the number of each tooth type was fortunately distributed similarly in each group, it seems that it could not be a source of bias. Regarding the follow-up radiographs, statistically significant decrease in the median of PAI score was observed in both groups, although the decrease in NaOCl group was significantly higher .This finding was in line with our previous antimicrobial survey which illustrated a more efficient reduction in post-treatment bacterial load of NaOCl group.

Considering the 30-month follow-up period, the PAI score for NaOCl group was significantly lower than IKI group. In other words, the healing process has completed faster in NaOCl group. Perhaps, the antimicrobial treatment and the reduction in bacterial colony count had altered the bacterial virulence properties but could not entirely render them harmless. From this point of view, it can be suggested that the higher incidence of residual microorganisms before root filling might defer, rather than stop, the healing process of the cases in IKI group.

Although in the first stage of the study, some cultivable bacteria were present in post-treatment samples of both groups (12); one hundred percent of the cases were healed, including those with positive cultures at the end of treatment. This result is almost consistent with the findings of several previous studies. Weiger et al. (36), Molander et al. (37) and Tervit et al. (32) showed that positive or negative cultures form the root canal will not significantly affect the healing results. Furthermore, Peters and Wesselink (19) demonstrated that the CFU < 102 at the time of obturation may not alter the outcome of treatment. These consequences can be referred to the fact that while the absence of bacteria might be a promising factor; it is the interaction between the host defence, the environment and the microbial pathogenicity that ultimately determines the development or resolution of apical periodontitis. Thus, while it is impossible to eradicate all root canal microorganisms with current techniques and materials, it is important to reduce their quantity to the optimum level of host immune system compatibility (37, 38).

The finding of present study may also emphasize the role of the root filling materials which might reduce the number of residual microorganisms. The antimicrobial effects of gutta-percha and Tubli-Seal sealer have been described by some authors previously (39-41). Moreover, root canal obturation may disturb the ecology of remaining microorganisms in the root canal system. This process may occur by entombing and depriving the bacteria from the nutrients and obscuring the required space for their reproduction (42).

The literature is lack of enough clinical evidences for the outcome assessment of using final rinse with IKI. The only available report is by Molander et al. comparing one-visit endodontic treatment of the teeth with apical periodontitis by NaOCl irrigation followed by IKI final rinse and two-visit treatment by applying calcium hydroxide (37). Their results did not represent the significant differences between the healing of groups (65% vs. 75%). In our study, we found that 100% of the cases were healed. The higher incidence of periapical healing in present survey can be attributed to this reason that the selected samples were all single-rooted teeth. As these teeth have less anatomical complexities in their root canal system, they are less challenging in disinfection procedures and may show better healing outcome when compared with multi rooted teeth (43). On the other hand, comparable results of healing rate in one visit endodontic treatments of single-rooted teeth with apical periodontitis have also been reported (32). Future research with more accurate techniques for detecting apical periodontitis, such as cone beam computed tomography, may need to confirm the study results.

## 5. Conclusion

This clinical trial demonstrated that root canal irrigation with NaOCl, resulted in a significant higher decrease in PAI scores in comparison to irrigation with normal saline followed by IKI final rinse. However, one hundred percent of the treated cases using NaOCl irrigation or normal saline followed by IKI final rinse were healed after the 30-month follow-up. In summary, although the culturing technique is a helpful measure to assess the effectiveness of antimicrobial protocols at the end of treatment sessions, it may not be the only predictor of the outcome.
